# Experiences and needs regarding information on nutrition and nutritional supplements among oncology healthcare professionals: an explanatory sequential mixed-methods study

**DOI:** 10.1007/s00520-026-10966-7

**Published:** 2026-07-10

**Authors:** Iris EC Nagtegaal, Loes van Rijssen, Barbara S. van der Meij, Manon GA van den Berg, Anja JThCM de Kruif, Petronella B. Ottevanger, Floortje K. Ploos van Amstel

**Affiliations:** 1 Department of Medical Oncology, Radboud University Medical Center, Nijmegen, The Netherlands; 2 Department of Nutrition, Dietetics and Lifestyle, HAN University of Applied Sciences, Nijmegen, The Netherlands; 3 Department of Human Nutrition and Health, Wageningen University & Research, Wageningen, The Netherlands; 4 Department of Gastroenterology and Hepatology-Dietetics, Radboud University Medical Center, Nijmegen, The Netherlands; 5 Department of Epidemiology and Datascience, Amsterdam University Medical Center Location VUmc, Amsterdam, The Netherlands

**Keywords:** Cancer, Experiences, Healthcare professionals, Nutrition information, Nutrition needs, Nutritional supplements

## Abstract

**Purpose:**

This study aimed to acquire a comprehensive understanding of the experiences and needs of healthcare professionals (HCPs) in a university medical center at the medical oncology department regarding the provision of uniform information on nutrition and nutritional supplements to adult patients with cancer.

**Methods:**

This explanatory sequential mixed-methods study employed an online questionnaire that was sent to HCPs and included questions on indications for providing nutrition advice, knowledge about nutrition and nutritional supplements, and needs regarding providing uniform advice about these topics. This was followed by semi-structured interviews with HCPs to gain deeper insights into their experiences and needs.

**Results:**

Thirty-two HCPs completed the questionnaire, and nine HCPs participated in semi-structured interviews. The results identified five themes: knowledge of nutrition and nutritional supplements, interdisciplinary care, patient-centered care, information sources, and (scientific) basis of nutrition information and research.

**Conclusion:**

This study shows that HCPs express a need for enhanced knowledge and greater consistency in nutritional care during cancer care. Although HCPs reported confidence in their general nutrition knowledge, they were less familiar with nutritional supplements. Education, awareness of guidelines, access to evidence-based information, a clearer division of roles between HCPs, and interprofessional collaboration are key to improving confidence, quality, and delivering personalized nutritional support in cancer care.

**Supplementary Information:**

The online version contains supplementary material available at 10.1007/s00520-026-10966-7.

## Introduction

Malnutrition affects up to 80% of patients with cancer and can lead to serious consequences, including impaired functional status, diminished quality of life, and poor survival [1–7]. This underscores the importance of adequate nutrition, nutritional information, and optimal nutritional guidance for patients with cancer throughout their cancer trajectory [2, 5, 8]. Cancer itself and its therapy frequently lead to appetite loss, nausea and vomiting, diarrhea or constipation, and fatigue, all of which reduce nutrient intake and contribute to weight loss [3–5].

Healthcare professionals (HCPs), such as nurses, physicians, and paramedical professionals (e.g., dietitians), play an important role in identifying and discussing nutritional problems during patients’ cancer trajectories. Adequate nutrition can help reduce treatment-related side effects in patients with cancer and enhance the strength and energy levels essential for tolerating and recovering from systemic cancer treatment, while also supporting the immune system [6, 9].

Multiple studies have indicated that patients with cancer, their partners, and relatives report that they do not receive sufficient information regarding nutrition and nutritional supplements. Due to this lack of information, they are frequently unaware of the available evidence-based information on nutrition and nutritional supplements [10–12]. In this paper, the term “nutritional supplements” refers to vitamins, minerals, and herbal preparations, and explicitly excludes oral nutritional supplements, such as energy- and protein-dense drinks used in management of malnutrition. Van Veen et al. [13] concluded that HCPs in the Netherlands considered themselves to be insufficiently informed and confident in providing nutritional advice. Similarly, previous research by de Kruif et al. [14] showed that while HCPs acknowledge the importance of nutritional issues, their knowledge, attitudes, and communication about nutrition vary considerably between professionals and settings. Furthermore, Murphy et al. [15] also noted that awareness of guidelines among HCPs varied, which impacted consistency in practice and patient care improvement.

The lack of high-quality evidence regarding the benefits and risks of nutritional supplement use is one of the factors contributing to the inconsistency and absence of nutritional guidance from HCPs [16]. Zirpoli et al. [17] investigated the provision of nutritional supplement recommendations to patients with cancer and demonstrated that 51% of patients did not receive recommendations from HCPs regarding supplement use.

Together, these findings highlight the urgent need to further involve HCPs in providing guidance and information on nutrition to patients with cancer. To improve cancer care, it is important to investigate HCPs’ experiences and needs concerning the provision of nutrition information during cancer trajectory. This study aimed to acquire a comprehensive understanding of the experiences and needs of HCPs regarding the provision of uniform guidance on nutrition and nutritional supplements to adult patients with cancer. To complement these findings, a parallel qualitative study was conducted to explore patients’ perspectives, wishes, and needs regarding this important topic (van der Meij et al., unpublished data, 2026).

## Methods

### Design and study population

This study used an explanatory sequential mixed-methods design, in which a quantitative phase was followed by a qualitative phase [18]. In this design, the qualitative phase was used to further explain and contextualize the results from the quantitative phase [18].

The study population consisted of HCPs from the medical oncology department of a university medical center, including (oncology) nurses, clinical nurse specialists, physician assistants, residents, and medical oncologists.

### Data collection

Data collection took place between April 2022 and May 2023.

#### Quantitative data

A digital questionnaire was distributed to HCPs. The questionnaire consisted of five sections: (1) demographic data; (2) frequency and indication(s) for providing nutritional advice to patients; (3) experiences and knowledge regarding the current advice on nutrition and nutritional supplements; (4) needs; (5) the extent to which they perceived or experienced a standardized approach in providing recommendations on nutrition and nutritional supplements in their medical oncology department (Appendix 1). The questionnaire included both closed and open-ended questions. Filtering questions allowed the HCPs to skip questions that were not applicable to them. Content validity was assessed by a panel of four experts (i.e., nursing scientists, clinical nurse specialist, and teachers from the [blinded for peer review]) in cancer care. They evaluated whether the instrument adequately represented the construct domain being measured [18].

#### Qualitative data

The semi-structured interviews were based on an interview guide incorporating topics derived from both the literature and insights obtained from the quantitative phase of the study (Appendix 2). Subsequently, the guide was reviewed, revised, and approved by a panel of three experts (FP, LR, IN). The topics included experiences related to providing nutritional advice, evidence-based practice, collaboration, and perceived needs. A pilot interview with a clinical nurse specialist was conducted to test the interview guide. The data collected from this pilot interview were not included in the results.

Semi-structured interviews were conducted with HCPs who met the inclusion criteria. Participants were selected through purposive sampling. To ensure a diverse range of experiences and needs from distinct roles, the principle of differentiation was applied by intentionally selecting HCPs from various professional roles (oncology nurses, clinical nurse specialists, physician assistants, medical residents, and medical oncologists).

The interviews, with a duration of 30–40 min, were facilitated by pairs of trained nursing students. Each interview was audio recorded using digital equipment.

### Data analyses

#### Quantitative data

Quantitative data were analyzed using IBM SPSS Statistics software, version 25.0 (IBM SPSS Statistics, Armonk, NY). Descriptive statistics were used to describe the demographic data. The results of the closed-ended questions were processed as numbers and percentages. Responses to the open-ended questions were used to complement the interpretation of the closed questions. The answers were compared and grouped into descriptive data.

#### Qualitative data

A thematic analysis was conducted as described by Braun and Clarke [19]. This analysis enabled researchers to explore and understand the complexity and nuances of qualitative data to gain profound insights into the experiences, perspectives, and behaviors of individuals [19].

Each interview was transcribed verbatim by four trained students and anonymized before being imported into a qualitative data management program (ATLAS.ti for Windows, V 8.0). All transcripts were reviewed by three researchers (FP, LR, IN) and concepts and ideas for coding were developed. Subsequently, the interviews were assigned to the three researchers. Each interview was coded by at least two researchers. After coding each interview, a consensus meeting was held to resolve discrepancies in coding through discussion until consensus was reached (FP, LR, IN). Themes and subthemes were generated through an iterative process, including discussions with the research team (FP, LR, IN, BM, MB). The Consolidated Criteria for Reporting Qualitative (COREQ) studies was used as guidance to report this study (Appendix 3)[20].

### Data triangulation

This study employed methodological triangulation using a quantitative method to initially assess HCPs’ experiences and needs regarding nutrition and nutritional supplements, followed by a qualitative method [21]. Integration was performed at the interpretation stage, where findings from both phases were compared and combined to provide a comprehensive understanding of HCPs’ experiences and needs regarding nutrition and nutritional supplements. Quantitative results were used to identify general patterns, while qualitative findings were used to explain, expand upon, and contextualize these patterns. Consistent with Moon’s [21] view of triangulation as a strategy to enhance validity, reliability, and legitimation, both datasets were compared to identify similarities, differences, and complementary insights. This integration process formed the basis for the overarching themes described in the results section.

### Ethical considerations

Ethical committee approval was not required for this research, as the study involved no medical interventions or risks to HCPs and was not covered by the Dutch WMO (Medical Research Involving Human Subjects Act). The questionnaires were anonymous. The interview recordings were securely erased after transcription, and the transcriptions were anonymized. The HCPs agreed to participate in the study by signing an informed consent form. This study was conducted in accordance with the EU GDPR (General Data Protection Regulation) [22, 23].

## Results

### Quantitative data

The questionnaire was distributed to 96 HCPs who met the inclusion criteria, of whom 32 (33%) completed the questionnaire. The study population consisted of three males and 29 females, and most of them were (oncology) nurses (*n* = 21, 66%). The respondents’ demographic characteristics are reported in Table 1. In total, 16 (50%) HCPs indicated that they provide information or guidance on nutrition, 2 (6%) focused on nutritional supplements, and 14 (44%) HCPs gave information on both topics to patients.

**Table 1:** Demographic characteristics of HCPs (*N* = 32)

Characteristics	*n* (%)
Age, median (range), y	43 (25–65)
Sex Male Female	3 (9) 29 (91)
Profession (Oncology) nurse Clinical nurse specialist Resident Medical oncologist	21 (66) 3 (9) 2 (6) 6 (19)
Department Nursing department Outpatient clinic Day care unit	12 (38) 16 (50) 4 (13)
Years of experience 0–5 y 6–15 y 16–25 y > 25 y	16 (50) 7 (22) 4 (13) 5 (16)

*HCPs* healthcare professionals, *n* number, *y* years

### Qualitative data

Nine HCPs participated in qualitative interviews. Among them were one physician assistant, two medical oncologists, one medical resident, four oncology nurses, and one clinical nurse specialist.

### Findings integration quantitative and qualitative data

**Fig. 1:**
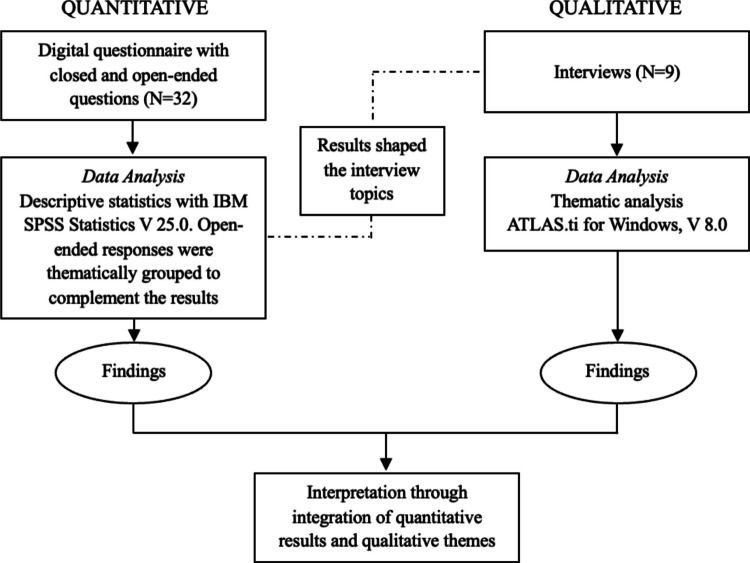
Flowchart of integration process of the quantitative and qualitative phases

Through the comprehensive merging and integration of quantitative and qualitative data, five themes were identified: knowledge of nutrition and nutritional supplements, interdisciplinary care, patient-centered care, information sources, and (scientific) basis of nutrition information and research. An overview of the integration based on quantitative and qualitative data is given in Table 2. Details of the integration process are shown in Fig. 1.

**Table 2:** Integration of quantitative and qualitative data

Themes	Quantitative data	Qualitative data	Integration
Knowledge of nutrition and nutritional supplements	Twenty-five (78%) HCPs reported having sufficient knowledge about nutrition and felt competent to deliver appropriate guidance	The interviews showed that most HCPs reported having adequate (basic) knowledge about nutrition “Well, I do have quite a bit of general knowledge about nutrition in cancer, particularly in terms of the advice we provide to patients with cancer.” (Respondent 7)	Convergence: both quantitative and qualitative findings indicate that HCPs feel competent in providing general nutritional advice, primarily at a basic level
	Twenty-five (78%) HCPs reported wanting to gain more knowledge, indicating a need to strengthen their general understanding for nutritional supplements	Several HCPs expressed a desire for a comprehensive overview of what is allowed in relation to specific types of systemic cancer medication “So, a wish for perhaps an overview of nutritional supplements and a wish that you might receive information [regarding developments of nutrition and nutritional supplements] once a year.” (Respondent 6)	Expansion: while quantitative data indicate a need for additional knowledge, qualitative findings further specify this need, particularly regarding the safe use of nutritional supplements in relation to cancer treatment
Interdisciplinary care	Regarding nutrition information, 14 (44%) HCPs experienced a uniform working method within their department Regarding nutritional supplements, 25 (78%) HCPs reported that they did not experience a uniform working method	Most of the HCPs interviewed indicated that they collaborated with other disciplines when providing advice on nutrition and nutritional supplements For example, some HCPs reported referring patients to a dietitian when nutritional inquiries became specific, when a patient had complex nutritional issues, or when energy requirements needed to be calculated	Divergence: although collaboration is reported in qualitative data, quantitative findings indicate a lack of uniformity in practice, particularly regarding nutritional supplements
	Providing advice about nutrition and nutritional supplements was offered by all HCPs (*n* = 32, 100%), and they considered this part of their professional responsibility	Opinions varied regarding who is responsible for providing advice on nutrition and nutritional supplements. The HCPs expressed a need for clarity regarding who holds which responsibilities	Expansion: while quantitative data suggest shared responsibility, qualitative findings reveal uncertainty and variation in perceived roles, particularly regarding nutritional supplements
Patient-centered care	About half of the HCPs (*n* = 17, 53%) reported receiving patients’ inquiries about nutrition and nutritional supplements on a weekly basis	The initiative for asking questions about nutrition and nutritional supplements primarily lay with the patients, according to the HCPs “The question often comes from the patient, especially from the younger generation.” (Respondent 9)	Convergence: both data sources indicate that patient inquiries are frequent and largely patient-initiated
	N/A	HCPs noticed that patients themselves wanted to do everything they could to combat cancer and maintain control, ensuring that they were aware of all developments related to nutritional supplements “There are quite a few people who are looking for alternatives and are involved with supplements.” (Respondent 6)	Silence: this theme emerged only from qualitative data, highlighting patient motivations that were not captured in the quantitative phase
Information sources	The information sources HCPs consulted to stay informed about the nutrition and nutritional supplements advice included (national) scientifically based websites about nutrition and nutritional supplements, and questioning colleagues	HCPs indicated that they also made use of each other’s knowledge by seeking advice from dietitians, pharmacists, a taste physiotherapist, interdisciplinary discussions, and scientifically based articles	Expansion: qualitative findings provide additional detail on how HCPs use and combine multiple information sources in practice
Scientific basis of information and research	N/A	Most HCPs expressed a need for scientific evidence that is particularly focused on nutritional supplements when providing advice to patients, to ensure that their recommendations are safe and effective “I want to provide advice on things that are scientifically substantiated and have medical content.” (Respondent 1)	Silence: the need for scientific evidence emerged only in the qualitative data and was not explicitly captured in the quantitative phase
	N/A	HCPs would appreciate a general, substantiated, evidence-based overview of nutritional supplements that patients may or may not take during their systemic cancer treatment “A general overview that I can easily look up. Sometimes, people use specific herbs like turmeric… and I have to check each herb individually to see if it can be safely combined with their chemotherapy. I find that challenging; it would be much easier if this information was more readily available.” (Respondent 4)	Silence: qualitative findings highlight practical needs for accessible evidence-based resources not identified in the quantitative data

*HCPs* healthcare professionals, *N/A* not applicable

Definition integration: convergence: (a) results of two phases directly agree; (b) expansion: one dataset provides additional detail or explanation; (c) divergence: findings differ between datasets; and (d) silence: themes only arising from the study of one phase

#### Knowledge of nutrition and nutritional supplements

Twenty-five (78%) HCPs reported having sufficient knowledge about nutrition and felt competent to deliver appropriate guidance, while at the same time 20 (63%) HCPs also expressed a desire to further expand their understanding in this area. The interviews showed that most HCPs reported having adequate (basic) knowledge about nutrition.

“Well, I do have quite a bit of general knowledge about nutrition in cancer, particularly in terms of the advice we provide to patients with cancer.” (Respondent 7)

In the context of advising on nutritional supplements, 25 (78%) HCPs reported wanting to gain more knowledge, indicating a need to strengthen their general understanding. In addition, 27 (84%) HCPs expressed the need to expand their existing expertise, for example, through clinical training sessions or patient case discussions.

One of the challenging factors identified by HCPs when providing advice on nutritional supplements was the wide variety of patient questions and the diversity of supplements used.

“For me it is more difficult when people use various supplements, and I am not exactly sure what is in them. I find that difficult sometimes, so sometimes I ask for the label of the product or request that people bring it with them so I can check it.” (Respondent 3)

HCPs expressed the need to provide clear advice on whether a food product or nutritional supplement is permitted and whether it can be used safely. Several HCPs expressed a desire for a comprehensive overview of what is allowed in relation to specific types of systemic cancer medication.

So, a wish for perhaps an overview of nutritional supplements and a wish that you might receive information [regarding developments of nutrition and nutritional supplements] once a year. (Respondent 6)

Several HCPs indicated that they knew where to seek information and who to consult regarding nutrition and nutritional supplements when their knowledge was insufficient. Nonetheless, they were eager to expand their knowledge in several ways.

“(…) inviting a dietitian would be nice, and we could then save up our questions about what they would advise in certain situations.” (Respondent 1)

#### Interdisciplinary care

Providing advice about nutrition and nutritional supplements was offered by all HCPs (*n* = 32, 100%), and they considered this part of their professional responsibility. Regarding nutrition information, 14 (44%) HCPs experienced a uniform working method within their department. They perceived this uniformity as clear in their internal communication as discussions among colleagues revealed no discrepancies in the advice provided to patients. HCPs who did not experience a uniform working method (*n* = 18, 56%) reported that a wide range of varying and changing advice was provided to the patients. Regarding nutritional supplements, 25 (78%) HCPs reported that they did not experience a uniform working method. They experienced limited alignment on this topic among HCPs, which resulted in a lack of awareness of each other’s approaches and advice.

Most of the HCPs interviewed indicated that they collaborated with other disciplines when providing advice on nutrition and nutritional supplements. For example, some HCPs reported referring patients to a dietitian when nutritional inquiries became specific, when a patient had complex nutritional issues, or when energy requirements needed to be calculated.

“In general, I think I manage quite well. When things get more specific, I do need a bit more support.” (Respondent 7)

The topics of nutrition and nutritional supplements were not routinely discussed among HCPs. Nutritional topics were occasionally addressed in case discussions, such as when initiating interventions like enteral feeding, or when a relevant study on nutrition is highlighted.

“Well, not specifically about nutrition. But if there are questions, we can discuss it. We do have collaborative moments with colleagues, but not about nutrition. No, it is somewhat of a neglected topic.” (Respondent 8)

“(…) that I would prefer having more conversations about this with oncologists and dietitians. I would actually have a need for that.” (Respondent 6)

The HCPs indicated that they were not always aware of which information about nutrition or nutritional supplements their colleagues had shared with their patients.

“I think we assume that we are roughly saying the same thing and using the same approach.” (Respondent 6)

Opinions varied regarding who is responsible for providing advice on nutrition and nutritional supplements. The HCPs expressed a need for clarity regarding who holds which responsibilities. Regarding the provision of nutritional advice, HCPs (including nurses) believe that this responsibility lies with nurses.

When it came to nutritional supplements’ advice, the situation became more complex. The responsibility of providing advice about nutritional supplements was perceived differently by the HCPs. Nurses indicated that they provide little or no advice about this and are not always sure who is responsible:

“And that becomes a bit of a point of discussion: is this the responsibility of the physician, or perhaps of others?” (Respondent 6)

“The oncologist prescribes chemotherapy, so they should also say whether it is allowed to use turmeric or CBD oil or whatever, yes.” (Respondent 5)

#### Patient-centered care

About half of the HCPs (*n* = 17, 53%) reported receiving patients’ inquiries about nutrition and nutritional supplements on a weekly basis. Most HCPs (*n* = 25, 78%) noted that they were usually able to answer these questions. The initiative to discuss nutrition and nutritional supplements with patients varied among HCPs. Specifically, nine (28%) HCPs never or rarely initiate such discussions; 14 (44%) sometimes do, and nine (28%) usually or always do. Examples of patient inquiries about nutrition include raw food, alcohol, sugar-free products, protein, and healthy eating. Questions about nutritional supplements include herbs, vitamins, fish oil, turmeric, and cannabis oil.

HCPs reported that patient-related factors, such as the type of cancer, stage of the disease, and emotional well-being, contribute to the process of providing information about nutrition and nutritional supplements.

“(…) in a care pathway involving stomach, intestinal, and liver cancer, there are numerous issues in the stomach, intestinal, and liver areas, so it is indeed a very significant challenge.” (Respondent 5)

The initiative for asking questions about nutrition and nutritional supplements primarily lay with the patients, according to the HCPs. Several HCPs reacted to patient questions.

“The question often comes from the patient, especially from the younger generation.” (Respondent 9)

Some HCPs have incorporated the provision and inquiry of information about nutrition and nutritional supplements into their care.

“But I do ask as standard how things are with the taste and appetite and if they have dry mucous membranes. And if I get a signal that things are not going well or I see the weight slowly decreasing, those are the moments when you give such advice.” (Respondent 2)

In addition, HCPs noticed that patients themselves wanted to do everything they could to combat cancer and maintain control, ensuring that they were aware of all developments related to nutritional supplements.

“There are quite a few people who are looking for alternatives and are involved with supplements.” (Respondent 6)

#### Information sources

The information sources HCPs consulted to stay informed about the nutrition and nutritional supplements advice included (national) scientifically based websites about nutrition and nutritional supplements, and questioning colleagues. Training sessions, guidelines, and scientific literature were also mentioned. Additionally, during the interviews, they indicated that they also made use of each other’s knowledge by seeking advice from dietitians, pharmacists, a taste physiotherapist, interdisciplinary discussions, and scientifically based articles.

“I often find it on https://www.wkof.nl/leven-met-kanker/voedingenkankerinfo/ [a Dutch scientific website about nutrition and cancer], where I can usually find the information.” (Respondent 4)

“…and we have a mailbox where we [HCPs] can ask questions [about nutritional supplements] to the pharmacists.” (Respondent 1)

“(…) because I literally learned it by occasionally attending a colleague's consultation to hear how he or she explained it.” (Respondent 3)

#### Scientific basis of information and research

Scientific evidence was not highlighted in the questionnaire’s responses; however, during the interviews, most professionals emphasized the importance of providing advice. Most HCPs expressed a need for scientific evidence that is particularly focused on nutritional supplements when providing advice to patients, to ensure that their recommendations are safe and effective.

“I want to provide advice on things that are scientifically substantiated and have medical content.” (Respondent 1)

Most HCPs indicated that they advise against using nutritional supplements when their effects and potentially harmful interactions with systemic cancer treatment had not been scientifically studied.

“So, try to avoid as much as possible doing anything that is not evidence-based.” (Respondent 8)

Due to changes and developments in cancer care and nutritional supplements, it is difficult to keep track of everything. In addition, according to some respondents, there is limited evidence either supporting or discouraging the use of nutritional supplements.

“There is minimal regular advice about supplements.” (Respondent 1)

HCPs would appreciate a general, substantiated, evidence-based overview of nutritional supplements that patients may or may not take during their systemic cancer treatment.

“A general overview that I can easily look up. Sometimes, people use specific herbs like turmeric… and I have to check each herb individually to see if it can be safely combined with their chemotherapy. I find that challenging; it would be much easier if this information was more readily available.” (Respondent 4)

## Discussion

This study explored the experiences and needs of HCPs regarding the provision of uniform guidance and information on nutrition and nutritional supplements to adult patients with cancer. Five key themes were identified: knowledge of nutrition and nutritional supplements, interdisciplinary care, patient-centered care, information sources, and (scientific) basis of nutrition information and research.

Overall, HCPs reported having sufficient knowledge about nutrition and expressed a desire to further expand their understanding in this area. In contrast, their knowledge about nutritional supplements was limited. This limited knowledge regarding nutritional supplements is due to the lack of scientific research on the effects of these supplements, especially during systemic cancer treatment. Although some knowledge gaps and needs regarding nutrition and nutritional supplements were identified among HCPs in this study, our parallel study (van der Meij et al., unpublished data, 2026) showed that patients generally expressed confidence in their HCPs’ nutritional expertise and relied on them for guidance and managing nutrition-related symptoms when needed. Despite the trust that patients place in their HCPs, challenges remain regarding the consistency of information provision on nutrition and nutritional supplements, partly because nutrition is not a standard topic of discussion for every HCP. Approximately half of the HCPs reported that there is no uniform approach to providing nutritional information during cancer care. Additionally, some patients indicated that they received conflicting advice regarding nutrition and nutritional supplements, which may contribute to feelings of uncertainty or doubt. At the same time, they expressed a clear need for consistency and clarity in the information provided (van der Meij et al., unpublished data, 2026). Murphy et al. [15] concluded that strengthening HCPs’ confidence in providing evidence-based advice on both nutrition and nutritional supplements is essential for promoting greater consistency in nutritional care practices. However, this raises the question of how HCPs can be supported to gain knowledge and become more consistent in providing nutritional information and advice?

Several studies have concluded that education could increase HCPs’ knowledge of nutrition and nutritional supplements [2, 13, 15]. This includes training in areas such as nutritional advice for specific types and stages of cancer, nutritional status assessment, alternative dietary approaches, and nutritional supplements [15]. This will lead to improved knowledge and consistency, which is also essential for delivering patient-centered nutritional care desired by both HCPs and patients (van der Meij et al., unpublished data, 2026) [7]. Besides, HCPs seek guidelines to support their care practices, as these provide a foundation for scientific evidence that enhances consistency and quality. Murphy et al. [15] found that the use of guidelines varied among HCPs, highlighting the need for more uniform adherence. Both education and guidelines were identified by the HCPs in our study to support the provision of consistent, evidence-based nutritional care.

HCPs also stressed the demand for scientific evidence, particularly regarding the use of nutritional supplements during cancer treatment. In contrast, patients are aware of the importance of scientific evidence but clearly pose that they would like more attention to complementary and alternative medicine in the hospital (van der Meij et al., unpublished data, 2026). However, this is a complex and often controversial issue. While some supplements may offer benefits in specific contexts, such as zinc supplementation which reduces the severity of oral mucositis during chemoradiation[24], other studies have linked supplement use to adverse outcomes. For instance, Smith et al. [25] and Mathijssen et al. [26] have shown that the use of St. John’s Wort may reduce the efficacy of chemotherapy and strongly recommended avoiding St. John’s Wort during systemic cancer treatment. These findings highlight the inconsistent and context-dependent nature of the evidence, which complicates the development of clear clinical guidance and poses challenges for HCPs seeking to provide individualized nutritional advice. This complexity emphasizes the need for a comprehensive overview that accommodates varying clinical scenarios.

To address the reported needs and experiences of HCPs, nutritional care during cancer treatment should be delivered through an interdisciplinary approach, as it requires the combined expertise of various HCPs in oncology practice. Previous studies have emphasized that nutritional support and the use of supplements are the most effective when integrated into multidisciplinary cancer care [2, 6, 8, 10]. Although our findings support the value of a multidisciplinary approach, they also reveal a lack of clarity regarding which HCPs are responsible for providing guidance on nutrition and supplement use. To improve the quality and consistency of nutritional care, clear role delineation and proactive integrated support should be prioritized within multidisciplinary oncology teams.

### Study strengths and limitations

A key strength of this study is the use of a mixed‑methods design, which enabled both the quantification of experiences and a deeper understanding of the underlying reasons behind them. The integration of quantitative and qualitative data provides a comprehensive and nuanced perspective on how HCPs address nutrition and nutritional supplement-related issues in cancer care. In addition, the inclusion of different professional groups, including physicians and nurses, strengthens the relevance of the findings, and reflects the multidisciplinary nature of nutritional care in oncology.

Several limitations should be considered. This study involved nursing students as interviewers, who had limited research and qualitative interviewing experience. This may have influenced data collection, depending on how the questions were asked. However, their involvement may also have contributed to a relatively open and unbiased interview setting.

The response rate of the questionnaire (33%) introduces a risk of non‑response and self‑selection bias. HCPs with greater interest in nutrition may have been more likely to participate, potentially leading to an overestimation of knowledge levels and perceived consistency in current practice. As a result, the findings may not fully reflect the perspectives of all HCPs involved in cancer care. Furthermore, because the questionnaire was completed anonymously, it is not possible to determine whether individual respondents also participated in the interviews. Although the same group of HCPs was invited for both components of the study, the extent of overlap between the quantitative and qualitative samples remains unclear, which may limit the interpretability of the findings. Finally, as this study was conducted in a single center, transferability is limited, and the results should be interpreted as context‑specific insights rather than broadly generalizable conclusions.

### Implications for practice

A complex issue in addressing all of the above needs lies in the shifting dynamics of contemporary healthcare. To complement our findings, patient perspectives were also explored in a parallel qualitative study among patients with cancer (van der Meij et al., unpublished data, 2026). As a result, a comprehensive understanding of both HCPs and patient perspectives was achieved. Patients increasingly wish to be actively involved in their care by sharing their perspectives and participating in decision-making. However, their views are often shaped by online content such as influencers and personal beliefs. This presents a challenge for HCPs, who must balance patient expectations with their professional responsibility to provide safe, evidence-based care [27]. This challenge is further intensified by the rapid pace of developments in oncology treatment, such as new types of systemic cancer therapies and the increasing attention to the use of nutritional supplements, making it increasingly difficult for HCPs to remain fully up to date. This raises important questions about the extent of knowledge that can reasonably be expected from HCPs, and where their responsibilities begin and end when advising patients on nutrition and supplement use. Artificial intelligence (AI) may offer valuable support by synthesizing evidence, identifying potential risks, and facilitating personalized guidance [28]. However, it should be noted that the reliability of this information depends on the sources utilized by AI.

## Conclusion

This study shows that HCPs express a need for enhanced knowledge and greater consistency in nutritional care during cancer care. Although general nutritional knowledge is well understood, nutritional supplements remain less familiar and are addressed with less confidence. Education, guideline awareness, and access to scientific evidence are crucial for enhancing HCPs’ knowledge. This not only improves their understanding of nutritional care, but also enables more effective communication with patients, leading to better-informed patients and personalized nutritional guidance. Additionally, the findings highlight the importance of mutual knowledge exchange among HCPs to reduce uncertainty. A clearer division of roles between HCPs, supported by education and interprofessional collaboration, may enhance the quality and personalization of nutritional care for patients with cancer.

## Supplementary Information

Below is the link to the electronic supplementary material.

ESM 1 (DOCX 39.6 KB)

ESM 2 (PDF 484 KB)

## Data Availability

No datasets were generated or analysed during the current study.
