# Supplementary material for: Experiences and needs regarding information on nutrition and nutritional supplements among oncology healthcare professionals: an explanatory sequential mixed-methods study
Source: Support Care Cancer. 2026 Jul 10;34(8):753. doi: 10.1007/s00520-026-10966-7 (PMC13354692; doi:10.1007/s00520-026-10966-7)
Supplement: Supplementary file 1 — (DOCX 39.6 KB) [file 520_2026_10966_MOESM1_ESM.docx]

**Appendix 1 – Questionnaire**

**Demographic data**

1. **What is your age?** Only numbers may be entered in this field.
2. **What is your gender?**

- Men
- Women
- Other

1. **What is your position within the Department of Medical Oncology?** Choose one of the following options:

- (Oncology) Nurse
- Clinical nurse specialist
- Physician Assistant
- (Oncology) Resident
- Medical Oncologist

1. **In which department are you primarily employed?** Choose one of the following options:

- Nursing department
- Outpatient clinic
- Day care unit

1. **How many years have you been working in the Department of Medical Oncology?** Choose one of the following options:

- 0–5 years
- 6–15 years
- 16–25 years
- 25+ years

**Questions about nutrition and nutritional supplements**

The following questions relate to nutrition and nutritional supplements. Tube feeding and medical nutritional drinks are not included here. Nutrition: Everything a person eats and drinks. Nutritional supplements: additional nutrients taken to supplement the regular diet. Examples include fish oil, vitamin C, magnesium, etc.

1. **Do you provide advice on nutrition and/or nutritional supplements?** Choose one of the following options:

- Yes
- No

*The survey closes if the respondent selects No*

1. **What do you advise patients with cancer about?** Choose one of the following options:

- Nutrition *(Questions 8 and 9 will appear next, thereafter question 12)*
- Nutritional supplements *(Questions 10 and 11 will appear next)*
- Both

1. **About which nutrition topics do you regularly receive questions?** Please name the 3 most common food items. [o*pen text field*]
2. **What kind of questions do you receive about these nutrition topics?** [o*pen text field*]
3. **About which nutritional supplements do you regularly receive questions?** Please name the 3 most common nutritional supplements*.* [o*pen text field*]
4. **What kind of questions do you receive about these nutritional supplements?** [o*pen text field*]
5. **When do you provide patients with advice on nutrition and/or nutritional supplements?** Select all options that apply to you:

- Before the start of treatment
- During treatment
- After the completion of treatment

1. **How often do you receive questions about nutrition and/or nutritional supplements from patients with cancer during treatment?** Choose one of the following options:

- Daily
- Weekly
- Monthly
- Rarely or never

1. **How do you experience patients’ questions about nutrition and/or nutritional supplements advice?** Choose one of the following options:

- Difficult
- Somewhat difficult
- Neutral
- Somewhat easy
- Easy

1. **To what extent do patients take the initiative to ask questions about nutrition and/or nutritional supplements?** Choose one of the following options:

- Never
- Sometimes
- Regularly
- Often
- Always

1. **To what extent do you take the initiative to provide advice on nutrition and nutritional supplements?** Choose one of the following options:

- Never
- Sometimes
- Regularly
- Often
- Always

1. **How do patients present their questions about nutrition and/or nutritional supplements?** Through…

- Patient contact at the bedside
- MijnRadboud
- (Medical) contact center
- An appointment at the outpatient clinic
- Contact with relatives
- The day treatment unit
- Other: *[open text field****]***

1. **What challenges do you encounter when giving advice on the use of nutrition and nutritional supplements by patients with cancer?** Multiple answers possible.

- Too little time
- Insufficient knowledge
- Uncertainty about giving advice
- Too little experience in giving advice
- I do not encounter any challenges
- Other: *[open text field]*

1. **Do you feel you have sufficient knowledge to provide education about nutrition to patients with cancer?**

- Yes
- No

1. **Do you feel you have sufficient knowledge to provide advice about nutritional supplements to patients with cancer?**

- Yes
- No

1. **Where do you obtain information to keep yourself updated on recommendations regarding nutrition and/or nutritional supplements?**

Please enter your answer here: *[open text field]*

1. **Do you have time during your working hours to maintain your knowledge about nutrition and nutritional supplement advice?** Choose one of the following options:

- Never
- Not often
- Sometimes
- Mostly
- Always

1. **Do you feel the need to expand your knowledge regarding nutritional advice for patients with cancer?**

- Yes
- No

1. **Do you feel the need to expand your knowledge regarding nutritional supplement advice for patients with cancer?**

- Yes
- No

1. **Do you feel the need for more time to stay updated on nutrition and nutritional supplement advice?**

- Yes
- No

1. **In what form would you prefer to deepen your knowledge on this topic?** Choose one of the following options:

- Conference
- Clinical lesson
- During the patient case discussion
- Training/education
- Personal time
- Other

1. **Do you feel there is a uniform approach in providing nutritional advice?**

- Yes
- No

1. **Please give an example that demonstrates that there is a uniform approach in providing nutritional advice.**

Please enter your answer here: *[open text field]*

1. **Please give an example that demonstrates that there is no uniform approach in providing nutritional advice.**

Please enter your answer here: *[open text field]*

1. **Do you feel there is a uniform approach in providing nutritional supplement advice?**

- Yes
- No

1. **Please give an example that demonstrates that there is a uniform approach in providing nutritional supplement advice.**

Please enter your answer here: *[open text field]*

1. **Please give an example that demonstrates that there is no uniform approach in providing nutritional supplement advice.**

Please enter your answer here: *[open text field]*

**Appendix 2 – Interview guide**

Introduction: You are being asked to participate in an interview study. First, thank you for taking the time to participate in this study. The aim of this study is to acquire a comprehensive understanding of the experiences and needs of HCPs regarding the provision of uniform information on nutrition and nutritional supplements to adult cancer patients. We will ask you questions, and you may choose not to answer at any time.

Some practical information:

- One researcher will conduct the interview, while another researcher will act as an observer, monitoring time and asking additional questions if necessary.
- We will adhere to the “Dutch Code of Conduct for Scientific Integrity” and ensure the confidentiality of your data.
- During the interview, you may indicate at any time that you wish to stop.
- The interview will be recorded using audio equipment.

If you agree, I would like to ask you to sign the “Informed Consent” document. When you sign this form, you are giving permission for us to use the information you provide during this interview.

Topic list

- Demographic characteristics
  - Function
  - Age
  - Profession
  - Department
  - Years of experience
- Experiences nutrition and nutritional supplement advice
  - How do you experience providing advice on nutrition and nutritional supplements?
  - How do you perceive questions related to nutrition and nutritional supplements?
    - About what?
    - When?
    - How?
    - Patient initiative?
    - Barriers
- Knowledge nutrition and nutritional supplement advice
  - How do you perceive the extent of your knowledge regarding nutrition? And what about nutritional supplements?
    - Source of information
    - Sufficient / insufficient knowledge
- Factors related to protocols and guidelines
  - Do you use protocols or guidelines when providing nutrition and nutritional supplement advice?
    - Which?
  - Other sources of knowledge
- Organizational factors
  - Is there any consultation or meeting within the department regarding the provision of advice?
    - Internal discussions
    - Uniform or non-uniform approach?
      - How?
    - Responsibility
    - Needs regarding nutrition and nutritional supplement advice

Is there anything else you would like to add or comment?

Thank you so much for your time and effort. You provided us with useful information.
